# A Survey of the Security Analysis of Embedded Devices

**DOI:** 10.3390/s23229221

**Published:** 2023-11-16

**Authors:** Xu Zhou, Pengfei Wang, Lei Zhou, Peng Xun, Kai Lu

**Affiliations:** College of Computer, National University of Defense Technology, Changsha 413000, China; zhouxu@nudt.edu.cn (X.Z.); pfwang@nudt.edu.cn (P.W.); cszhoulei@gmail.com (L.Z.); kailu@nudt.edu.cn (K.L.)

**Keywords:** embedded device, security analysis, fuzzing, emulation, static analysis, dynamic analysis

## Abstract

Embedded devices are pervasive nowadays with the rapid development of the Internet of Things (IoT). This brings significant security issues that make the security analysis of embedded devices important. This paper presents a survey on the security analysis research of embedded devices. First, we analyze the embedded device types and their operating systems. Then, we describe a major dynamic security analysis method for an embedded device, i.e., simulating the firmware of the embedded device and performing fuzzing on the web interface provided by the firmware. Third, we discuss some other issues in embedded security analysis, such as analyzing the attack surface, applying static analysis, and performing large-scale analysis. Based on these analyses, we finally conclude three challenges in the current research and present our insights for future research directions.

## 1. Introduction

With the rapid development of the Internet of Things (IoT) and modern smart devices, embedded computing systems that are small and cheap become ubiquitous. Embedded devices are applied in industry, education, and our everyday life. We can find embedded devices almost everywhere, such as WiFi routers at home, IP cameras in the streets, network printers at offices, traffic lights on roads, the control systems in modern cars, the smartwatch we wear, drones we fly, and even toys our children play with. As IoT Analytics estimated, there were 12.2 billion active IoT devices globally by the end of 2021. The number will grow by 18% to 14.4 billion by the end of 2022, and it is expected to be approximately 27 billion by 2025 [1]. The estimated number only includes the embedded devices that are connected to the Internet.

Embedded devices bring severe security problems to the Internet for three reasons [2,3]. First, the hardware resource of embedded devices is often limited, e.g., lower capacity of CPU, memory and power. Consequently, embedded software (often referred to as firmware, including an operating system and applications) is often tailored to suit the hardware. As a result, modern advanced security mechanisms (e.g., ASLR, DEP, Control-Flow Integrity) are often missing in embedded systems because they may take additional computational power and memory capacity, thus making embedded systems prone to attack. Second, embedded devices lack security updates. Many manufacturers do not provide security updates for their cheap products due to economic reasons. Even worse, users tend to not update their firmware as long as the device is functioning. Third, the number of embedded devices is especially large as we have discussed, which makes them very easy to reach.

As a result, attacks that target embedded devices are increasing. For example, the Mirai malware that affects IoT devices, especially DVR or IP cameras, can create a large botnet of as many as 50 thousand devices [4]. Recently, a vulnerability involving an out-of-bounds read was discovered in the module library of TPM2.0 [5]. Exploiting this vulnerability allows an attacker to access sensitive data stored in the TPM, potentially affecting billions of embedded devices. This issue is not limited to consumer devices, as demonstrated by a vulnerability revealed in Cisco ISE’s Embedded Service Router (ESR) by *CVE-2023-20193*. This vulnerability enables authenticated local attackers to read, write, or delete arbitrary files on the underlying operating system and escalate their privileges to root. The 2022 Microsoft Digital Defense Report [6] reveals that more than 10 critical vulnerabilities are present in 32% of firmware images, making embedded devices an increasingly popular target.

Therefore, it is important to study the security problem of embedded devices. Mahmoud et al. and Bures et al. publish two surveys discussing the security issues of IoT systems [2,3]. They analyzed the limits of the IoT hardware, emphasizing the importance and challenges of the IoT security and providing suggestions to enhance the IoT security and reliability from the development perspective. While both of them focus on the research on how to design security mechanisms or security principles for development, they miss the security analysis research that can be applied after the development procedure, i.e., when the embedded product is manufactured and delivered to customers. This survey focuses on this research, which is helpful in fixing vulnerabilities in commercial embedded devices and delivering patches.

Security analysis is useful to detect vulnerabilities of embedded systems and help manufacturers to fix them. The fix can be made before a product is launched on the market, or be shipped with security updates for end users. Therefore, research on security analysis for embedded systems is helpful for the security of the Internet. However, research in this field is very complex. They should deal with many issues due to the variety of embedded targets, such as (1) considering many types of hardware and operating systems; (2) using different kinds of analysis technologies, including code audit, fuzzing, symbolic execution, emulation, protocol analysis, etc., and (3) reaching specific goals like peripherals inference, bug detection, large scale analysis, etc.

To get a sense of the current progress in this research field and provide insights for future research directions, we surveyed to summarize the research on the security analysis of embedded devices. First, we conclude the hardware and operating systems of embedded devices. We adopt the categories made in [7]. Then, we survey the ideas and technologies used in security analysis. Many technologies are applied in the current research, and they can be divided into two major categories: static analysis and dynamic analysis. Static analysis will analyze the embedded systems—either the source code or the binary—without running it. On the contrary, dynamic analysis will test the embedded system by running it—either in hardware or in an emulated environment. In this paper, we mainly talk about a dynamic analysis method (the main method) using emulation to run the firmware and fuzzing the web interface to detect vulnerabilities. We further talk about optimizations of this method, such as how to increase the emulation success rate, how to generate valid test cases, and how to optimize the fuzzing throughput. Bug detection is a major problem in dynamic analysis due to the lacking of protection mechanism in hardware or OSes, which we will discuss in detail. Third, besides the main method, we talk about some other issues in the embedded security analysis: (1) we analyze the attack surface of embedded devices other than web interfaces; (2) we discuss how static analysis can be used to analyze embedded systems; (3) we reveal the purpose and method of performing large scale analysis. Finally, we conclude the three challenges of the current research and provide insights for future research directions.

There are two contributions from this survey. First, it studies and summaries the security analysis research of embedded devices so far, especially the dynamic and static analysis of the embedded firmware to detect vulnerabilities. This is helpful for beginners to learn this research field. Second, this paper presents the authors’ insights into challenges and future research directions based on the summary of the current research, which may help researchers to find new ideas.

The rest of this paper is organized as follows. Section 2 gives a brief introduction of embeded devices—their hardware and operating systems. From Section 3 to Section 6, we discuss the dynamic analysis methods for embedded devices. Section 3 introduces two different methodologies to run firmware. In Section 4, we talk about how to dynamically detect vulnerabilities in the web interface of embedded devices by using fuzzing. Section 5 discusses the bug detection methods of dynamic analysis. In Section 6, we discuss a possible attack surface in dynamic analysis other than the web interface. In Section 7, we introduce the static analysis method for embedded devices. In Section 8, we discuss how to perform a large-scale analysis for both dynamic and static analysis. After introducing the security analysis methods, we talk about the challenges and future research directions in Section 9. Finally, we conclude in Section 10 and list threats to validity in Section 11.

## 2. Embedded Device

An embedded device is a specialized computer system equipped with a small operating system or a single application running on it. An embedded device is often designed for one or very few specific purposes, e.g., controlling the traffic lights, driving a camera, printing pages on demand, doing WiFi routing, etc. Since the embedded devices are not designed for multiple purposes, the hardware is often tailored for an economic reason, e.g., using embedded CPU with low computing capability, using small on-chip memory, using small flash memory as storage, and using minimum I/O ports and peripherals. As a result, embedded OSes are also tailored for limited hardware. According to [7], embedded systems (embedded hardware plus embedded OS) can be divided into three categories.

Type I has full-functional hardware and a general operating system. The computing power is lower than desktop hardware, while important features (e.g., MMU) are integrated. The OS running on it is general purpose with modern desktop OS features, but tools and libraries are slimmed down to a small size to fit the hardware. A typical example of this kind is a home WiFi router. It is a small but is a whole computing system with, for example, ARM CortexM CPU, memory and flash storage, Ethernet and WiFi network, USB ports, etc. Most WiFi routers run the OpenWrt [8] operating system, which is a variant of Linux designed for embedded devices. OpenWrt uses a simplified toolset like busybox—the firmware is often as small as several MBs, but functions just like the Linux operating system. Similar operating systems include Android, Windows Embedded CE, RT-Thread IoT, Nucleus RTOS, etc. Vulnerabilities in Type I embedded systems are mainly related to the user applications and services provided by these devices. For example, these kind of embedded devices often have web pages for administrators to maintain the device. Typical vulnerabilities in such a system are like XSS, Command Injection, etc. Network services provided by such embedded systems such as DNS, Samba, etc. may also introduce overflow or configuration vulnerabilities.

Type II has more limited hardware resources than Type I. Though Type II still maintains an OS abstraction, the OS is not general purpose. The OS is used to ship several applications to perform a single task. The characteristic of a Type II system is that its hardware may lack advanced processor features such as a MMU. As a result, the applications running on such a system are usually highly coupled. Such operating systems include uClinux, ZephyrOS, and VxWorks [7]. Type III often works as a slave device such as a Bluetooth dongle, USB storage, PLC, etc. The hardware of this kind is often a single-chip microcomputer. Type III has no operating system abstraction, which means such an OS is just a library for development. More specifically, applications are integrated into the OS running directly on the hardware. Hence, the firmware of this kind of embedded system is often called monolithic firmware [7,9,10]. The corresponding OS for developing Type III systems include Contiki, TinyOS, mbed OS2 [7], ThreadX, eCos, RT-Thread nano, FreeRTOS, etc. Since embedded systems of Type II and Type III devices are simple binaries, vulnerabilities in such systems are often buffer overflows. These vulnerabilities are relatively easy to exploit because these systems lack security mechanisms. The exploit of these devices such as Bluetooth may be escalated to the host system (e.g., Android), causing the underlying host to be exploited (e.g., CVE-2019-11516) [11].

**Figure 1:**
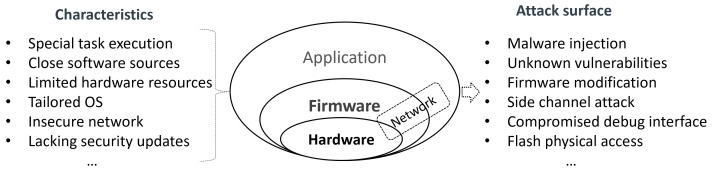
Security risks in embedded device system.

**Security risks.** There are more serious security risks in current embedded systems due to their distinct characteristics, as shown in Figure 1. Different privilege levels in an embedded system expose various attack surfaces. In embedded software, such as applications, operating systems, and firmware, attacks resemble those on general desktop or server OSes. An attacker can exploit design defects (*vulnerabilities*) in the platform to inject malicious code (*malware inject*) into the runtime system in different devices. In embedded hardware components, such as CPUs and memory, the design is often simplified to optimize cost and performance, resulting in a lack of protection. This allows attackers to bypass software protections and gain direct access to the hardware. For instance, an attacker can exploit the workflow of a process by accessing the CPU through a debugger channel (*compromised debugging interface*). Furthermore, the attacker can directly access specific memory, exploiting user-sensitive data through techniques like DMA, bus snooping, and cache buffer (*side channel attacks*). In a compromised embedded device, attackers can even install malicious firmware (*firmware modification or flash reinstall*), enabling them to launch attacker-designed applications on the device and provide erroneous services.

Despite the security threats of embedded devices, doing security analysis for these devices is not easy. The diverse hardware and software configurations make it difficult to create a general emulation environment for security analysis, especially for monolithic firmware. For example, monolithic firmware often needs to identify the entry point for firmware initialization [12] or dump the state of a running snapshot [11], which requires additional reverse engineering efforts and resources for execution dumping. Currently, firmware code and workflow are evaluated for security using dynamic and static analysis mechanisms. However, these mechanisms have become ineffective for several reasons. Dynamic analysis has become more challenging because of the increased difficulty in injecting malformed data or monitoring crashes and coverage. Similarly, static analysis has also become harder due to the increased coupling between the operating system and application code. Additionally, the increasing diversity of hardware and operating systems from Type I to Type III hampers security analysis at large scales. Thus, we will delve into the details of characteristics and security risks of embedded devices and analyze the advantages and disadvantages of existing firmware emulation approaches. This will provide an overview of how effective approaches can be designed for future research.

## 3. Dynamic Analysis: Emulation vs. Physical Device

In this section, we talk about how to perform dynamic analysis for embedded systems. To perform dynamic analysis, we have to run the target embedded system first. There are two major ways to do this: (1) using physical devices, and (2) using emulation.

It is straight forward to test the physical devices of an embedded devices. As for the emulation-based method, we take the OpenWrt firmware that is widely used in WiFi routers as an example to explain how to perform dynamic analysis. The dynamic analysis precedure can be divided into four parts. First, we unpack the firmware with tools like binwalk. Since the OpenWrt firmware is often packed in a SquashFS format, we should first extract the file system from the firmware to get its kernel, binaries, configure files, scripts, etc. Then, we repack the file system with our debug tools into a Qemu disk image. Third, we emulate the firmware by running a Qemu instance attaching the Qemu disk image. Finally, when the firmware is correctly emulated, the network and the web service would work. Hence, we can perform a web fuzzing tool such as boofuzz to mine the vulnerabilities of the firmware’s web interface. It is possible to find vulnerability types such as XSS, buffer overflow and command injection.

We compare the typical characteristics of the hardware-based method and the emulation-based method in Table 1.

### 3.1. Why Emulation

One way to perform dynamic analysis is by using the hardware device, e.g., turning on the power of an embedded device and fuzzing it through the network. Snipuzz [13] is a blackbox fuzzer that can test hardware devices directly. It sends mutated messages (test cases) to the target device and monitors the responses. It improves mutation efficiency by inferring the protocol message format (message snippet) from the responses. Similar research includes IoTFuzzer [14], which fuzzes IoT devices through mobile APP management interface and WMIFuzzer [15] which fuzzes commercial off-the-shelf (COTS) IoT devices through web management Interface.

The other method of dynamic analysis is based on software emulation instead of a hardware device. This method first extracts firmware from the embedded device or downloads it from the manufacturer’s website. Then, it uses a system emulator (usually QEMU [16]) to run the firmware. Finally, it performs dynamic analysis (usually fuzzing) on the emulated environment [17]. Many research studies follow this methodology, e.g., Firmadyne [17], FirmAE [18], FirmFuzz [19], FirmHunter [20], FirmAFL [21], etc.

The hardware-based method is easy to implement, but it has many drawbacks. First, the hardware-based method is slower than the emulation-based method [7,21]. This result seems counter-intuitive since software emulation is faster than hardware. The reason is that the hardware-based method uses embedded CPU which is often much slower than a desktop CPU, and emulation is usually carried out on a desktop CPU. Hence, even degraded by emulation, the performance of desktop CPU is still higher than that of the embedded CPU.

Second, it is hard to monitor the tested devices in embedded hardware. Dynamic analysis such as fuzzing wants to know the status of the device under test (DUT), e.g., whether a crash is triggered, the current coverage information, etc. However, as the hardware resource of the embedded device and its OS are often limited, it is not trivial work to use mechanisms of instrumentation, debugging, etc. to monitor the running status of the DUT. As a result, the hardware-based method often falls into blackbox fuzzing, which uses response messages to identify the liveness of the DUT [13,15]. Even worse, this method can be invalid due to the silent memory corruptions, which were studied by Muench et al., who point out memory corruptions in embedded systems may not be observed [7].

Third, it is a financial burden to acquire and maintain as many embedded devices. With every device tested, we have to buy it, which will cost money as there are many hundreds of thousands of embedded device types and their numbers are growing very fast. Even if we have enough financial support, sooner or later, we will have our library full of all kinds of embedded devices, which will need a special human resource to maintain. That does not include the network we have to construct and the electrical power we have to supply. Finally, it is not easy to scale the testing capability for the hardware-based method. For example, if we want to speed up the testing procedure for 10×, we have to buy the same kind of embedded device for 10. Moreover, we have to set up the testing environment 10 times as well.

The emulation-based method, on the other hand, does not have the above problems at all. Emulation is faster than embedded hardware due to the power of a desktop or server-side computers. It is easy to monitor the status of the running firmware thanks to the emulators [22,23,24,25,26]. We only have to acquire the firmware of an embedded device—which is often available on the website of the manufacturer—to perform security analysis. And finally, it is easy to scale the analysis by simply duplicating multiple emulation instances. The only difficult part of the emulation-based method is to successfully emulate the hardware, and researchers are continuously making progress in this field [9,10,11,17,27,28,29,30]. We foresee that the emulation-based method will be the dominating method for dynamic analysis.

### 3.2. General Emulation Methodology

Emulation can be divided into user-model emulation and system-mode emulation. User-model emulation only emulates a user-level application, and relays the host system to perform system calls. Typical user-model emulation tools include Pin [31] which only emulates x86 instructions, and user-mode QEMU [16] which supports most common CPU architectures such as x86, ARM, MIPS, PowerPC, RISCV, etc. On the other hand, system-model emulation will emulate a whole computer system and run an entire operating system on it, including user applications. System-mode emulation is like virtualization, except that each instruction is emulated by a set of host instructions instead of directly being executed on the host hardware. This causes two consequences: (1) emulation is usually much slower than virtualization, and (2) emulation may support heterogeneous architectures between guest and host. System-model emulation that emulates both CPU and peripherals is called full-system emulation. Typical full-system emulation tools include system-mode QEMU, Simics [32], Bochs [33], etc. Unicorn [34] is a special system-model emulator that only emulates a CPU without peripherals; thus, it is often referred to as a CPU emulator.

Intuitively, system-model emulation is suitable for analyzing firmware, as the firmware is a whole operating system running on an embedded device. However, this leads to a trade-off when we just want to analyze the specified application in the firmware, e.g., the web service. Costin et al. compare different levels of emulation and conclude that system-mode emulation is still the best choice, even for analyzing user applications [35]. The reason is that user applications often rely on the underlying OS environment of the firmware. Without the OS environment, user applications are difficult to start.

In our testing process, we have encountered some challenges when trying to emulate firmware images in a full-system emulation environment. Out of the 6000 firmware images we tested, approximately half of them had file systems and were able to be emulated. However, only around 1000 of these images (about 1/6 of the total) could be successfully loaded in the QEMU environment and accessed through the web interface, as shown in Table 2. The first challenge we faced in full-system emulation is related to the underlying operating system (OS) present in the firmware. The OS often contains specific configurations and drivers for different peripherals. This poses a difficulty when trying to emulate the original OS kernel of the firmware in the emulation environment. The second challenge is the performance impact of emulating system calls and other unrelated processes. Full-system emulation can be resource-intensive and result in significant performance costs. Emulating system calls and managing unrelated processes further adds to this overhead. These challenges highlight the complexities involved in efficiently emulating firmware images in a full-system emulation environment.

Firmadyne [17] solves the first problem by using a manually crafted Linux kernel to substitute the original kernel in the firmware. They write dummy drivers for NVRAM that are used in most embedded systems so that the manually crafted kernel can read NVRAM-related configurations. They inject a console application at the early booting stage to print the kernel’s booting information for debugging. They provide three such kernels to support multiple architectures. They even instrument system calls to infer the network configuration for automated configuring of the network. Based on their work, FirmAE [18] further investigates many emulation failures and introduces several heuristics to improve the emulation success rate.

Another way to solve the peripheral emulation problem is by using mixed emulation. Avatar [25,26] set up two running instances: the physical device instance and its emulation instance. It orchestrates the two running instances at runtime. Normally, dynamic analysis is performed on the emulation instance. When emulation encounters I/O access, it forwards the access to the physical device and returns data to the emulation instance. PROSPECT [36] uses a similar approach—it forwards accesses to peripherals to real peripheral hardware to facilitate emulation.

The second problem is studied in Firm-AFL [21], which uses a mixed emulation, i.e., using user-mode emulation and system-model emulation correspondingly to improve performance. They maintain two running instances. Normally, the user-mode emulation runs, which is fast. When it encounters a system call, it switches to the system-mode emulation and switches back when the system call is finished. By using this method, they increase the testing throughput from several test cases per second to hundreds of test cases per second. Unfortunately, this kind of execution is prone to incur inconsistency. Meanwhile, the deploying cost is high as a complex system is applied to coordinate the two running instances and the source code is not available.

### 3.3. Re-Hosting

The above emulation approaches can help to emulate Type I embedded devices and perform security analyses for user applications in firmware. However, in order to analyze the original OS kernel or embedded firmware of Type II or Type III, we have to turn to the technology of re-hosting. Re-hosting literally means to re-host a firmware from the actual hardware to an emulated hardware. Therefore, the firmware should remain unchanged, which is a base requirement to analyze the original OS kernel in Type I embedded devices or firmware binaries in Type II and Type III embedded devices.

For a general OS, the key to re-hosting is to deal with peripherals, making the peripheral divers in the original OS kernel work with the simulated peripheral device model. FirmGuide [29] leverages symbolic execution to infer the device models of peripherals like interrupt, timer, and UART. Specifically, it performs symbolic execution on the device driver to guess the state transfer model of the device. By using the state transfer model, it automatically generates a QEMU device model implementation using C code. The QEMU device model will emulate the peripheral hardware and talk to the device driver. The opposite way to make device drivers cooperate with emulated hardware is by modifying device drivers instead of building device models. ECMO is such a work [28]. ECMO instruments the device drivers with unimplemented peripherals and makes them cooperate with standard peripherals, or just work as a dummy node without crashing the entire OS.

HALucinator [9] aims at Type III embedded devices with monolithic firmware that usually has no OS abstraction. The authors learned that monolithic firmware usually adopts the Hardware Abstraction Layer (HAL) to ignore hardware detail. Hence, they leverage the HAL mechanism to implement device models for peripherals. To this end, they compile the HAL code to binaries. Then, they use libMatch—a binary comparison tool—to compare firmware binaries with HAL binaries to locate HAL code stubs in firmware. Finally, they replace the HAL code stubs and implement corresponding device models to make them cooperate. Frankenstein [11] demonstrates how to re-host the firmware of a single SoC chip with itself being peripheral. Frankenstein re-hosts the Broadcom Bluetooth firmware by dumping the running status, memories, and codes into a snapshot file, which is then compiled into an ELF binary. The ELF binary is emulated by a user-mode QEMU and connected to the Bluetooth protocol stack in the host by using btproxy.

uEMU [37] operates in two phases to emulate firmware with unknown peripherals. Firstly, it performs a knowledge extraction phase to build a knowledge base on how to respond to peripheral accesses and identify the data registers used for I/O operations. Secondly, it employs dynamic analysis techniques to test the firmware. Once rehosted in the emulator, uEMU identifies vulnerabilities in the firmware code to assess device security.

Fuzzware [10] introduces a pattern-based MMIO modeling approach to trace MMIO (Memory-Mapped Input/Output) accesses directly. It focuses on re-hosting firmware without relying on coarse-grained static models of hardware behavior or involving manual effort. Compared to uEMU, Fuzzware achieves improved results by automatically reducing the input overhead of MMIO modeling, which includes minimizing manual efforts, incomplete overhead elimination, and path elimination.

sEMU [38] addresses the challenge of lacking peripheral models during firmware emulation. It builds peripheral models using a natural language processing (NLP) approach, translating human language descriptions of peripheral behaviors into structured condition-action rules. The key advantage of sEMU is its ability to dynamically synthesize a peripheral model for each firmware execution. However, the accuracy of the peripheral model is contingent on the limitations of NLP tools regarding handling references across different knowledge.

Note that many re-hosting approaches primarily focus on emulating firmware based on the Arm Instruction Set Architecture (ISA). However, with the deployment of IoT devices featuring various CPU architectures such as Arm, ×86, Power PC, MIPS, etc., Jetset [39] aims to address this diversity by emulating three different architectures. To enable support for a broader range of firmware, it is crucial to extend current emulator tools such as QEMU or modified versions to encompass more CPU architectures. This flexible approach allows researchers to broaden the scope of firmware emulation with investments in software development. In summary, the mentioned re-hosting approaches contribute to the field by addressing specific challenges in emulating firmware, such as building knowledge bases, reducing input overhead, synthesizing peripheral models, and expanding support for various CPU architectures.

## 4. Fuzzing Web Interface

Most embedded devices, such as WiFi routers, IP cameras, and Network Attached Storage (NAS), usually provide a web interface to users for maintenance, making the web interface a major attack surface to hackers. This also draws the researcher’s attention—dynamic analysis of the web interface of embedded devices is studied by many researchers [17,19,20]. The web of the embedded device is usually different from the web on the Internet. The latter is used by end users to maintain their data, e.g., browsing a web page, downloading or uploading a picture, modifying personal profile, etc. However, the former is used by the device owner to maintain the configuration of the device. The web actions are usually turned into commands executed by the device. Therefore, this kind of web is often more prone to Command Injection (CI) vulnerability. Moreover, security transmission such as HTTPS is often invalid on these webs [17]. Even if HTTPS is enabled, it would be useless due to the non-trust of self-signed certification. Due to the short time window for market launching, web pages in embedded devices are often not well designed, leaving many XSS vulnerabilities [40,41].

### 4.1. General Methodology

The naive way to fuzz the web interface of the embedded device is like this: use a headless web browser to retrieve web pages and manipulate items such as inputs, links, and buttons, fill the input with random strings and submit it to the server [17,42,43,44,45], and monitor the liveness of the server by using response message. This method is simple but inefficient, which is only useful when no other ways are available.

**Figure 2:**
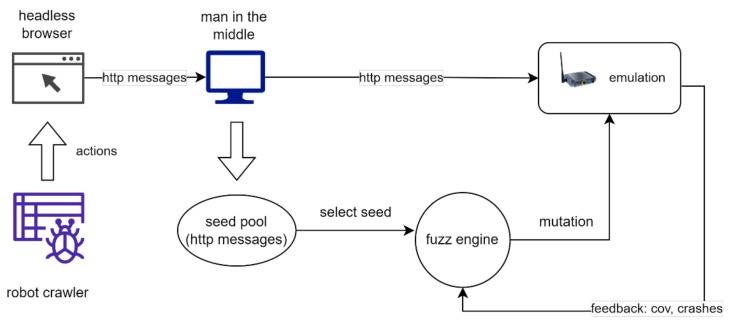
The general architecture of web fuzzing for embedded system.

The state-of-the-art fuzzing architecture for embedded web interface [19,20,46] is shown in Figure 2. A middle man is set up between the headless browser and the server. The man in the middle can be implemented by using network proxy tools like w13scan or mitmproxy. The headless browser requests web pages from the server, while a robot crawler is responsible for crawling and manipulating the web pages to trigger network traffic. Meanwhile, the man in the middle collects the network messages as initial fuzzing seeds. After initial fuzzing seeds are collected, a fuzzing engine mutates the seeds to generate malformed messages (test cases) and sends them to the DUT. Meanwhile, it monitors the crashes to report Buffer Overflow (BO) vulnerabilities and gathers coverage information as feedback to direct the next round of mutations. CI vulnerability is detected by injecting certain command strings, e.g., touch filename, and checking if the command is executed, e.g., the specified file is created. The XSS vulnerability is detected similarly by examining logs to check whether the injected JavaScript codes are executed.

### 4.2. Optimization

One issue that should be noted is that although HTTP is a stateless protocol, web applications usually maintain a state between messages. For example, a user should first make an authentication before performing further actions. The authentication and the following messages become stateful messages which have a dependency. Two other dependencies are caused by (1) cookies and parameters passed through messages, and (2) a message field is dependent on another field. The stateful messages should be identified and grouped according to their dependency. To generate valid test cases, one should mutate a group of ordered messages together and preserve the message dependency [20].

**Graybox fuzzing**. Graybox fuzzing leverages feedback such as coverage information to guide the test case generation, i.e., selecting a high-priority seed for mutation according to coverage increment [47]. However, it is not trivial work to get the coverage information for embedded systems as monitoring means are invalid due to resource limitations, as we have discussed in the previous section. However, by using emulation, we can get adequate feedback for graybox fuzzing. This can be done by using QEMU-based tools. For example, DECAF [22,24] can be used to identify running processes in an emulated OS (Linux or Windows), while PANDA [23] can help us to get the path coverage of a running process. Path coverage is sensitive but costly. It may be a better choice to use more coarse-grind coverage feedback. For example, FirmHunter [20] uses three kinds of coverage feedback at different granularities to balance performance and accuracy, including path coverage, function coverage, and system-call coverage.

**Static analysis**. Static analysis can be used to facilitate web fuzzing of embedded systems. IoTParser [46] performs static analysis on firmware. First, it scans the firmware’s root file system to identify web pages (html, php, jsp, etc.) and CGI executables. Then, it analyzes the web pages and CGI executables to infer possible URLs (web interfaces), parameters, and shared keywords. The inferred URLs and parameters are used to supplement the initial seeds that are missing or even cannot be reached (in the case of the hidden interface [48]) by the crawler, while shared keywords are used to prioritize the seeds (seeds with shared keywords will have higher priority). Static analysis optimization is useful as it eliminates some non-determinism for dynamic analysis.

**Throughput**. The above optimizations all focus on how to generate more valid test cases, i.e., they are trying to make every single test more meaningful. However, the testing throughput is also a bottleneck for embedded systems. The hardware-based fuzzing will issue around one test case per second. For the emulation-based method, the throughput is only increased to several test cases per second. This is much lower compared to fuzzing the desktop applications, which are usually thousands of test cases or more per second [49]. Firm-AFL [21] analyzes the throughput problem of the emulation-based method, and points out that full-system emulation incurs overhead due to (1) memory address translation; (2) dynamic code translation; and (3) system call emulation. To improve fuzzing throughput, it proposes augmented process emulation (APE), which uses user-mode emulation to boast performance and uses full system emulation to ensure correctness.

## 5. Bug Detection

Bugs (or vulnerabilities) can be divided into Overflow type and Logical type. The overflow type usually causes crashes of the target processes or the whole operating system, except for the silent memory corruption situations [7]. Nevertheless, Overflow bugs are still detectable by using response messages or logs. The response message is a simple way to check the liveness of a blackbox executing instance. When the fuzzer issues a message, if the DUT does not respond or responds with an unexpected message, we can conclude that the DUT incurs a bug. This simple method is often used for testing embedded hardware when no other monitoring measures are valid.

Another way to check the overflow bugs is by examining the logs for crash information. Many kinds of logs can be used, e.g., the dmesg kernel log of Linux, the log of the Apache or nginx server, etc. We can also make our own log in the emulator by writing QEMU plugins just as PANDA and DECAF do. The silent memory corruption detection can be mitigated by building log patterns for this kind of corruption [7].

However, there is not a general method to detect logical bugs, such as Command Injection, XSS, and Authentication Bypass (AB). Command Injection bugs can be detected by mixing specified command strings into mutated test cases and checking if the command is executed. XSS bugs can be detected in a similar way by checking whether the injected JS code is executed. To detect authentication bypass, one has to identify which parts should be authorized to access. For web applications, it is easy to identify—almost all web pages except the main page and the error report pages should be authorized to access. IoTScope [48] scans all the web pages in the root file system of the firmware and tests if the pages can be accessed without authentication. It further proposes to use frequency statistics to check whether a page is an error report page or not. By doing so, it can automatically report whether an accessed page is an authentication bypass.

However, it is not easy to detect authentication bypass for binaries. One has to identify which part of the code is a privileged operation, and the privileged operation can be executed without authentication. Firmalice [12] uses heuristics to identify privileged operations. Then it builds an input determinism model to identify authentication bypass—if there is exactly one path from input to the privileged operation, it is a backdoor. Firmalice uses symbolic execution to compute possible paths from input to privileged operation.

## 6. Attack Surface

**Mobile APP**. Besides web interface, embedded devices have other attack surfaces which should be tested. With the development of smartphones, many manufacturers adopt apps to control and configure IoT devices for convenience. For example, the Canon Inkjet printer for home and small offices provides APP to end users to set up the printer and print documents. IoTFuzzer [14] studies the communication channel between APPs and IoT devices and acts as an APP to send malformed messages to IoT devices for fuzzing.

**Network protocol**. Embedded devices also provide many network services at the application level, which enlarge the attack surface accordingly. For example, an NAS device will act as a file station, thus providing Samba service; a WiFi router will provide DHCP and DNS services; and an IP camera will provide video streams. These application-level network services can be analyzed by a network protocol fuzzing tool, e.g., boofuzz [50], Peach [51], Kitty [52], etc., as long as the protocol model has been built. For unknown protocols, the typical method is to collect network traffic to reverse-engineer the protocol model first, and then perform a protocol fuzzing based on the generated protocol model [53,54]. This reverse-engineer method is not mature but has proved to be useful in non-complex protocols of industrial control systems [55].

IoT-dedicated protocols should be paid much attention to. MQTT is such a protocol that is widely used in IoT devices to transmit messages between devices, update firmware, and notify the current status to the cloud. We foresee testing of MQTT will be a hot research field for the security analysis of embedded devices as it becomes more and more widely used.

**Wireless communication** is also an important attack surface for embedded systems. This includes Bluetooth, WiFi, Zigbee, z-wave, etc. Wireless communication is usually implemented by an SoC (controller) equipped with an antenna. The SoC runs an independent small OS on it, which is responsible for processing data between the host computer and the air: (1) it receives data from the antenna, unpacking it, and then repacking it for the host OS; (2) it processes the message sending requests from the host, preparing data packets and calling the modem to send the information through the antenna to the air. Wireless communication may have vulnerabilities in the firmware of the SoC or the host drivers that talk to the SoC. A typical dynamic security analysis method for wireless communication is to inject data from the air and check crashes in the controller and the host.

Frankenstein performs dynamic analysis for the Broadcom Bluetooth chip [11], which is a combo chip for both Bluetooth and WiFi. They dump the snapshot of the running chip to make an ELF executable. Then, they emulate the chip by using a user-mode QEMU. They inject data by analyzing the function symbol of the firmware and instrument procedures for receiving data from the antenna. Frankenstein aims to fuzz the controller, not the host, although vulnerabilities in the controller may cause crashes of the host. SweynTooth [56], on the other hand, fuzzes the controller and the host as a whole. They first study the Bluetooth specification to build a protocol model. They use this model to generate valid Bluetooth packets (test cases), mutate them, and send the malformed packets using a Bluetooth dongle to the tested device with Bluetooth through the air. The malformed data will enter the tested device through its antenna, then to the Bluetooth chip, and finally to the host operating system. However, the drawback of this method is that if a crash occurs, one cannot identify whether it happens in the Bluetooth controller or the host. InternalBlue [30] is a dynamic analysis tool for the Bluetooth chip. It is capable of analyzing, monitoring, and patching the Bluetooth chip. These functions of InternalBlue are used to build Frankenstein [11].

**MMIO**. For small embedded devices with monolithic firmware (e.g., smart meters, PLCs), there are no common interaction interfaces such as network or wireless communication. But, they are still equipped with sensors to acquire data. These sensors act as peripherals to the embedded systems and become the attack surface. Fuzzware [10] works out a way to fuzz this kind of embedded device. They focus on the MMIO used by the device to configure and exchange data with the peripherals. They use symbolic execution to guide the mutation to generate valid MMIO accesses and mutate the MMIO access data to fuzz the device firmware.

## 7. Static Analysis

In this section and the next, we talk about two important issues in the security analysis of embedded systems: static analysis and large-scale analysis. Besides dynamic analysis, static analysis is another option for finding vulnerabilities in embedded devices. Static analysis is performed on the firmware of embedded systems. Therefore, it has the same problem with the emulation-based analysis method—they have to get the firmware first. These researchers either use a crawler to get the firmware from the Internet, or work out a way to extract firmware from the device hardware by using the serial port, JTAG, or flash programmer. After getting the firmware, they have to extract the root file system by using tools like binwalk [57], firmware-mod-kit [58], dji-firmware-tools [59], etc. The root file system is used to perform static analysis.

Static analysis can be performed at the source code level [60], or the binary level [61,62]. Software component analysis and homology analysis [63] can be used to identify software modules that contain known vulnerabilities in the firmware. The Firmware Analysis and Comparison Tool (FACT) [64] is an open-sourced static analysis tool for firmware. It is a framework that integrates a lot of plugins to analyze the firmware, such as extracting the arch information, performing sensitive data leak checking, comparing to the vulnerability database (e.g., CVE) to detect known vulnerabilities.

Costin et al. performed the following security analysis on firmware: (1) they cracked the hashed password files to find weak passwords, (2) they performed correlation analysis to find leaked certifications, common keywords of backdoor, and known vulnerabilities of correlated files, and (3) they used data enhancement to find more information of the firmware using a search engine. In correlation analysis, the leaked certifications can be used to compromise online devices (found by ZMAP [65] that contain the leaked certifications. Correlation of files is detected by using fuzzy hash, e.g., ssdeep, sdhash [66].

## 8. Large Scale Analysis

Automated firmware analysis at a large scale is very important as embedded devices are ubiquitous and numerous. It is meaningful to have the analyzing speed exceed the increasing speed of embedded devices. Static analysis is easy to scale as there is no need to run the firmware before performing security analysis. The scalability for static analysis is generally limited due to the difficulty in acquiring closed firmware. Costin et al. propose a large-scale analysis using static analysis [66]. They used a crawler to crawl 284 sites and acquire 32 thousand firmware images. To collect closed firmware, they also provide a web submission interface for users to manually submit their own firmware. To scale the analysis, they used a private cloud composed of 90 computing nodes, producing 10 GB of analyzing results in the database.

However, it is non-travail work to perform dynamic analysis on a large scale. To dynamically analyze embedded devices, we have to make the analyzing procedure fully automated. For example, Firmadyne [17] will (1) automatically download and extract a firmware, (2) automatically identify the architecture of the firmware, (3) automatically make the QEMU image from the root file system of the firmware and a manually crafted Linux kernel, (4) automatically infer the network configurations as well as generate the running script, and (5) automatically run the emulation to perform fuzzing. Costin et al. propose a similar automated dynamic analysis framework at the same time [35]. The major difference is the way of emulation. Firmadyne changes the kernel of the emulated firmware and retains the root file system, while [35] boots a general Linux distribution (Debian Squeeze) and change-root to the root file system of the original firmware and re-executes the init scripts.

Nevertheless, the difficulty for large-scale dynamic analysis mainly lies in automated emulation. First, the diversity of embedded devices makes it impossible to emulate all kinds of firmware with only one general emulation method. We have to deal with many types of CPU architectures, operating systems, and peripherals—each new situation may cause manual effort to fix the emulation system. However, researchers are making progress in emulating Type I embedded systems that use general architecture and operating systems [18,19,21,35]. Second, the emulation of firmware costs a lot of computing energy which brings scalability limits for financial reasons. Third, the emulation for the original kernel or firmware images (re-hosting), especially for the monolithic firmware, is a known challenge (as discussed in Section 3) on the matter of making this process automatic.

## 9. Summary

So far, we have discussed various security analysis methods for embedded devices, including dynamic analysis, static analysis, emulation, and large-scale analysis. A summary and comparison of the typical research mentioned in this paper is shown in Table 3.

### 9.1. Challenges

The current research is making great progress in performing security analyses for embedded devices, but there are still limitations. Here, we conclude three major challenges in the security analysis of embedded systems that have not been fully addressed.

- Automated analysis. It is a challenge to make the security analysis of embedded systems fully automated. Manual effort is often required to extract firmware from hardware, select configurations for the operating system, and build a peripheral model for the device. All the efforts are spent in dealing with the diversity of embedded devices, which are their natural characteristic. On the other hand, automated analysis is so important because it is the basis of performing large-scale analysis, which is the key to saving security analysts from being exhausted.
- Throughput. The emulation-based dynamic analysis is limited by the testing throughput. Emulation is often one or two orders of magnitude slower than the original execution. This cannot be easily solved as we need the full-system emulation to retain the original executing environment for the firmware. However, this incurs a lot of unrelated code to be emulated, which degrades performance heavily. The current throughput is several test cases per second. Even improved by user-mode emulation, the throughput is several hundreds of test cases per second, which is still much slower than that of the desktop application fuzzing. One possible solution is to use virtualization. However, it only works when the guest and host have the same architecture.
- Re-hosting. Though several types of research have been published to solve this problem, re-hosting of embedded firmware is still a difficult problem. The difficulty lies in that we have to infer the hardware model through the firmware binary itself. Current researchers try to mitigate the information gap by assuming the firmware is using Hardware Abstraction Layer (HAL), or by using symbolic execution to guess the correct actions of hardware. They solve the problem in one or two situations, which is far from practical.

### 9.2. Future Work

In this subsection, we propose our insights for future research directions in the security analysis of embedded devices based on the challenges we analyzed and the new opportunities we have. We think future research will work in three aspects: static analysis, large-scale analysis, and re-hosting.

**Static analysis**. The advantage of static analysis is that analysis can be performed without running the firmware, and thus, it is fast and easy to use. Static analysis is rapidly developing in analyzing the desktop application, from source level to binary level [61]. Software component analysis and homology analysis [67,68] is an emerging technology to scan known vulnerabilities quickly in binary code by using a large vulnerability database. Modern technologies also study a way to perform code audits for binaries by automated decompiling binaries into source code [61]. The typical false-positive problem can also be mitigated by using a constraint solver. Furthermore, static analysis can also be used to facilitate dynamic analysis [46,48,69]. In short, static analysis should play a more important role in firmware security analysis in the future.

**Large-scale analysis**. The large-scale analysis is a very important feature to deal with the huge number of embedded systems. However, large-scale analysis is still a challenge due to the diversity of embedded systems, while the diversity is still increasing. One possible way to solve this problem is by building our knowledge of embedded systems, their CPU architectures, operating systems, normal configurations, peripherals models, etc. This is not a task for one researcher or two. Platform and protocol should be set up for all researchers to work on. Nevertheless, this is not an easy task, and future researchers should pay more attention to it according to our opinion.

**Re-hosting**. Emulation is still the major way to perform dynamic analysis, while re-hosting is the most difficult part of emulation. This is the key to analyzing embedded systems of Type III, which have wide use in control systems of industry such as PLCs, and important function chips such as Bluetooth, smart meter, hard drives, etc. The current research is not practical enough, leaving future researchers a long way to go.

## 10. Conclusions

In this paper, we study the current security analysis research of embedded devices. First, we introduce a taxonomy for embedded devices from a security perspective. This taxonomy divides embedded devices into three categories according to their capability in hardware and operating system, with each category having a different security level. We mainly discuss two different ways of security analysis—dynamic analysis and static analysis. We first introduce dynamic analysis, which is powerful but difficult. In dynamic analysis, firmware should be emulated first to bypass the limitation of the underlying hardware. Then, fuzzing can be used to mine vulnerabilities from attack interfaces such as Web interfaces. Several other attack interfaces and the bug detection methods are also discussed. Besides the dynamic security analysis, we also discuss the static analysis, which does not have to run (or emulate) the firmware. Compared to dynamic analysis, static analysis is simple and easy to use, but is not accurate. Dynamic analysis and static analysis are complementary in performing security analysis for embedded devices. Finally, we also discuss the approaches to extend security analysis to a large scale, i.e., performing dynamic analysis or static analysis on thousands of embedded firmwares. Based on our study, we conclude three challenges in this research field—the difficulties of making security analysis fully automated, increasing the testing throughput for dynamic analysis, and rehosting the firmware. We also propose three future research directions, which we think are important for solving the current challenges—static analysis, large-scale analysis and rehosting.

## 11. Threats to Validity

Due to the knowledge limit of the author and the rapid development of the research, some methods and literature may have been missed in this survey. Evidence given might be limited due to the extent of the paper. Future research directions discussed in this survey are also from the authors’ point of view, which may not be true.

## Figures and Tables

**Table 1:** Comparison of typical characteristics of hardware-based and emulation-based dynamic analysis.

Attributes	Hardware-Based	Emulation-Based
throughput	low	high
monitor & control difficulty	high	low
financial cost	high	low
scalability	low	high
technical difficulty	low	high

**Table 2:** Emulation evaluation with part of firmware images.

Vendors	Types	Test Cases	Succ. Retrieved	ISA: [Num]	Succ. Accessed	Avg. Size (MB)
ASUS	router bluetooth	1625	1432	Arm: [695] MIPS: [724]	713	31
Dlink	router camera	2246	674	Arm: [150] MIPS: [501] Intelel: [7] PPCeb: [15]	241	6.3
Netgear	router	1573	682	Arm: [353] MIPS: [292] Intelel: [7] PPCeb: [30]	73	11.3
Trendnet	router	604	221	Arm: [30] MIPS: [191]	73	9.11

**Table 3:** A list of research studies in security analysis of embedded systems. In Column 4, * means the research is not limited to a specified attack interface. In Column 5, ✓ means the research is applicable to large-scale analysis, while ✗ means the research is not applicable to large-scale analysis.

Project	Device Type	Related Method	Attack Surface	Large-Scale?
Firmadyne	I	dynamic/emulation/fuzz	web	✓
FirmAE	I	dynamic/emulation/fuzz	web	✓
FirmFuzz	I	dynamic/emulation/fuzz	web	✓
FirmHunter	I	dynamic/emulation/fuzz	web	✓
IoTFuzzer	I	dynamic/hardware/fuzz	app	✗
FirmAFL	I	dynamic/emulation/fuzz	network protocol	✗
Firmalice	I, II, III	static/emulation/SE	backdoor	✗
Fuzzware	III	rehosting	MMIO	✗
InternalBlue	III	rehosting	bluetooth	✗
Large-scale embedded	I, II, III	static	*	✓
Avatar& Avatar2	I, II, III	dynamic/emulation	*	✗
SwegnTooth	I, II, III	dynamic/hardware/fuzz	bluetooth	✗
IoTScope	I	static	web	✓
Frankenstein	III	rehosting	bluetooth	✗
Dynamic Analysis	I	dynamic/emulation/fuzz	web	✓
FirmGuide	I	emulation/rehosting	*	✗
ECMO	I	emulation/rehosting	*	✗
HALucinator	III	emulation/rehosting	*	✗
What you corrupt	I, II, III	emulation/bug detection	*	*

## Data Availability

Data are contained within the article.
